# A circuit model of the temporal pattern generator of *Caenorhabditis *egg-laying behavior

**DOI:** 10.1186/1752-0509-4-81

**Published:** 2010-06-07

**Authors:** Mi Zhang, William R Schafer, Rainer Breitling

**Affiliations:** 1 Division of Biology, University of California, San Diego, La Jolla, CA 92093, USA; 2 Department of Physics, Harvard University, Cambridge, Massachusetts 02138, USA; 3 Medical Research Council (MRC) Laboratory of Molecular Biology, Cambridge CB2 0QH, UK; 4 Groningen Bioinformatics Centre, University of Groningen, 9751 NN Haren, The Netherlands; 5 Faculty of Biomedical and Life Sciences, University of Glasgow, Glasgow G12 8QQ, UK

## Abstract

**Background:**

Egg-laying behavior in the nematode *C. elegans *displays a distinct clustered temporal pattern: egg-laying events occur primarily in bursts or active phases, separated by inactive phases during which eggs are retained. The onset of the active phase can be modeled as a Poisson process with a time constant of approximately 20 minutes, while egg-laying events within an active phase occur with a faster time constant of approximately 20 seconds. Here we propose a cellular model for how the temporal pattern of egg-laying might be generated, based on genetic and cell-biological experiments and statistical analyses.

**Results:**

We suggest that the HSN neuron is the executive neuron driving egg-laying events. We propose that the VC neurons act as "single egg counters" that inhibit HSN activity for short periods in response to individual egg-laying events. We further propose that the uv1 neuroendocrine cells are "cluster counters", which inhibit HSN activity for longer periods and are responsible for the time constant of the inactive phase. Together they form an integrated circuit that drives the clustered egg-laying pattern.

**Conclusions:**

The detailed predictions derived from this model can now be tested by straightforward validation experiments.

## Background

The execution of specific behaviors relies on specialized motor programs, which generate specific spatial and temporal patterns of muscle contraction. The regulatory circuits that control the patterning are in principle independent of the concrete instantiation of the muscular end organ (see for example [1]). The same logical regulatory structure can be used to control a wide variety of behavioral programs. This also means that studying such pattern generating circuitries in experimental organisms can provide insights into fundamental circuit designs that are re-used in the much more complex brains of higher animals. The neuronal circuits of the worm *C. elegans *are particularly attractive for this kind of basic studies, because all neurons and muscle cells are individually known, their connectivity is constant, and the transparent body facilitates visualization and manipulation of single cells in living animals. In addition, genetic manipulation is much easier than in vertebrates. In the present paper we take advantage of these experimental options to elucidate the logical connectivity of the circuit controlling the egg-laying behavior of *C. elegans *and show how it implements basic mathematic operations that control the temporal pattern of egg-laying.

**Figure 1:**
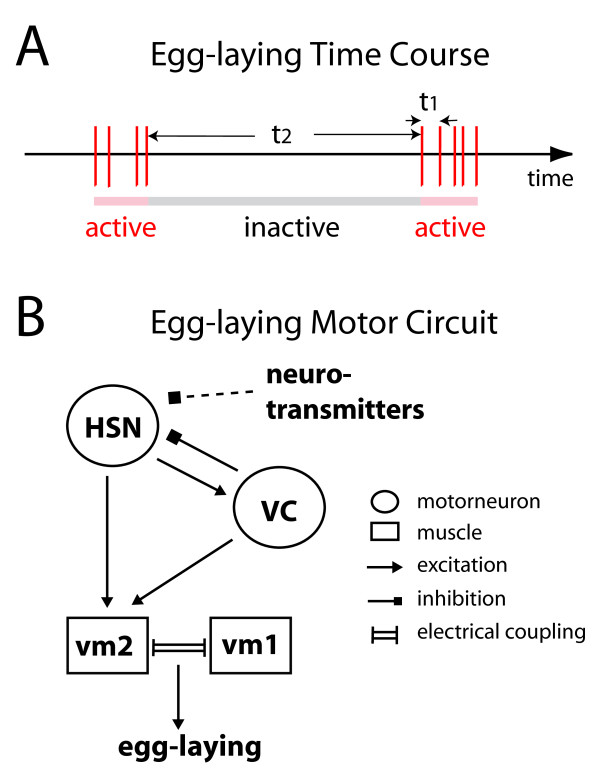
**Clustered temporal pattern of *C. elegans *egg-laying behavior and its neuronal control**. A) Cartoon representation of egg-laying behavior. Each red vertical bar indicates one egg-laying event; egg-laying shows a clustered temporal pattern: short hyperactive periods (pink) are interspersed by longer quiescent periods (gray). The egg-laying intervals within active states are termed intra-cluster interval (τ_1_); the intervals between the last event of one cluster and the first one of the next cluster covering an inactive state are termed inter-cluster interval (τ_2_). B) Neuronal circuit inducing individual egg-laying events. HSN autonomous spiking activity will activate vulval muscles (vm1 and vm2), both directly and redundantly through VC neurons. Vulval muscles are of two types, vm1 and vm2, and are connected by gap junctions. Their simultaneous contraction leads to transient opening of the vulva and a single egg being laid. VC has also been found to have an inhibitory effect on HSN.

*C. elegans *egg-laying occurs as a time series of stochastic events. In first approximation, egg-laying is a random point process: individual egg-laying events occur aperiodically and are sufficiently brief (<0.1 sec) that they can be treated as point events. However, it is clear that egg-laying is not strictly random; the time and location of egg-laying events is regulated by diverse environmental and internal factors, and under certain conditions egg-laying can be suppressed for long periods. The overall temporal pattern of egg-laying behavior is clustered [2]: short periods of high-frequency egg-laying behavior, called active states (2-10 minutes; about 1 egg/min), are separated by much longer quiescent periods about 20 minutes to 1 hour long, called inactive states. Figure 1A shows this pattern and also defines the related terminology. The intra-cluster time intervals (i.e. the time between two consecutive egg-laying events within the active state) are exponentially distributed, with τ_1_; ~20 sec in wild type worms. Likewise, the inter-cluster time intervals (the time between the last egg-laying event of one cluster to the first event in the next cluster) are exponentially distributed with a slower time constant τ_2_; ~20 min. The exact start and end point of the active and inactive states are impossible to determine, as the first and last egg-laying event of a cluster are close, but not exactly equal, to the transition between states. The uncertainty will be on the order of half an intra-cluster interval (0.5*τ_1_), much shorter than the duration of the states themselves. As Zhou et al. have shown [2], the clustered pattern can therefore be described by a combination of two Poisson processes with different time constants.

The core components of the neural circuit driving each individual egg-laying event have recently been characterized ([3]; Figure 1B). Two major pairs of neurons contribute directly to the control of egg-laying. The HSN neurons are the central command neurons of the circuit; each autonomous individual Ca^2+ ^spike in HSN can induce contraction of the vulval muscle cells (vm1/vm2) and result in one egg-laying event. The HSNs are modulated by the VC neurons, and also integrate environmentally-controlled neuromodulatory signals that influence egg-laying frequency. For example, posterior body touch transiently inhibits egg-laying by exciting the PLM mechanoreceptor neurons, which in turn directly inhibit HSN activity.

How do these cells create the clustered temporal pattern in egg-laying? Which cells are required to cause the regular transition between active and inactive states? In this paper we propose a model to explain this behavior using evidence from cell biology, genetics and statistics. Ca^2+ ^levels in the HSNs (as well as other neurons of the circuit) have been visualized at high temporal resolution using calcium-sensitive fluorescent dyes, giving us an opportunity to directly monitor the transmission and integration of signals in the circuit. Furthermore, mutants with defects in individual cells make it possible to discriminate correlated excitation from causal links in the circuit. Based on these data, we propose an integrated circuit model that can explain the logic underlying the egg-laying temporal pattern.

## Results

### HSN is under negative feedback regulation from egg-laying

**Figure 2:**
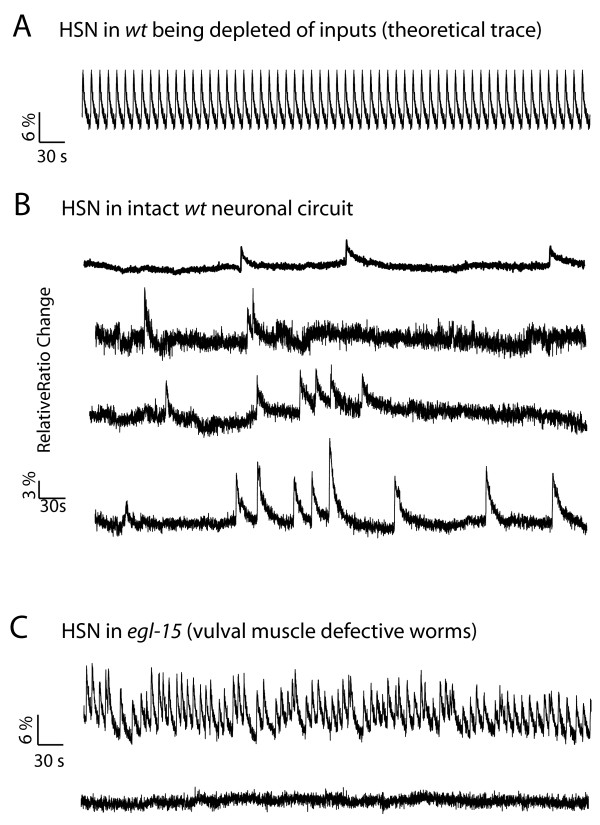
**HSN activity is subject to negative feedback modulation by egg-laying**. **A) **Without any modulations, HSN will be autonomously oscillating in Ca^2+ ^levels as shown by a theoretical cameleon trace. B) In a physiological ionic environment HSN activity shows sparse spikes, suggesting that it is under inhibitory modulation (four representative traces are shown). C) In *egl-15 *worms, where egg-laying is prevented due to abnormal vulval muscle development, HSN inhibition is partially removed, with neurons exhibiting unconstrained oscillations about half of the time, similar to the theoretical pattern in panel A. The other half of recordings are silent as indicated by the second trace.

It has been suggested that in the absence of external modulation, the HSNs would be autonomously oscillating [3] (illustrated in Figure 2A). This has been shown by separately depriving HSN of its synaptic or diffusible inputs. In comparison, HSN in the context of an intact neural circuit shows sparse spiking activity (Figure 2B), demonstrating that it is normally under negative regulation in physiological conditions.

Interestingly, in *egl-15(n484) *worms, which are defective in egg-laying behavior due to abnormal vulval muscle development, we found that half of HSN recordings exhibit unrestrained oscillations very similar to the theoretical pattern in Figure 2A, with HSN oscillating about half the time (4 out of 9 recordings) and silent otherwise (Figure 2C). The overall activity of *egl-15 *HSN is much higher than that of wild-type HSN, due to the hyperactive oscillations in the former. This suggests that egg-laying itself provides inhibitory feedback to the HSN neurons.

### Egg-laying provides inhibitory feedback to the VC neurons

**Figure 3:**
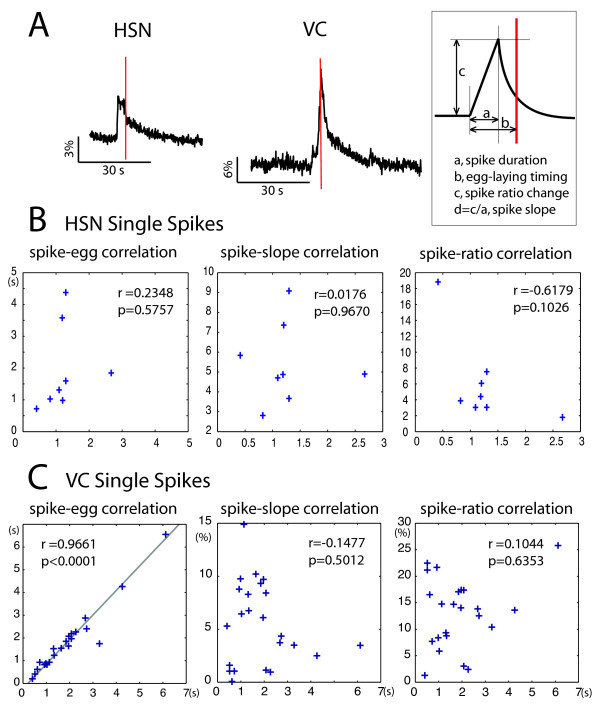
**Egg-laying correlates with repolarization in VC Ca^2+ ^spikes**. A) Representative Ca^2+ ^spikes in HSN and VC cells during egg-laying. The inset defines the terms used: a: spike duration is time from the start of depolarization until the peak of the spike. B): egg-laying timing is the time from the start of depolarization until the egg-laying event. c: spike ratio change is the background normalized cameleon ratio signal difference between the start of depolarization and the peak of the spike. d: the depolarization slope is c over a. B) and C) Correlation for various pairs of parameters in HSN and VC spikes, respectively. The Pearson correlation coefficient (*r*) and corresponding *p*-value are indicated. A strong statistically significant correlation is only found between egg-laying timings and VC spike timings.

Each egg-laying event is preceded by an autonomous depolarization of the HSN and VC neurons, observable as an increase of Ca^2+ ^levels in the cell ([3]; Figure 3A). The egg-laying motor program occurs very quickly, so egg-laying usually is finished well before the excited HSN and VC neurons come back to rest.

To determine the causal relationship between HSN activity and egg-laying muscle contraction, we asked if there is a fixed correlation between the timing of egg-laying and the HSN and VC calcium spikes. Specifically, we investigated whether egg-laying is coupled to any specific point during the calcium transient. A co-variation test (Figure 3B) showed that egg-laying happens when Ca^2+ ^levels are at their peak in the VC neurons. Linear regression shows that egg-laying and the calcium peak happen almost simultaneously, as the slope of the regression line is close to 1 and the intercept close to 0. Thus, egg-laying is tightly coordinated with the end of the calcium transient. From a mechanistic point of view this is surprising: as egg-laying is caused by VC depolarization (which leads to neurotransmitter release and vulval muscle contraction), we might expect egg-laying to occur at a constant delay after the beginning of the depolarization. In contrast, since the duration of the transient (from onset to peak) is highly variable, a correlation between egg-laying and the calcium peak was unanticipated.

As the spike peak reflects the start of repolarization, the simplest explanation for the coupling of egg-laying to spike peaks is that the repolarization of the VC neurons is actually induced by egg-laying. This would establish a negative feedback loop, where depolarization of VC causes egg-laying, which in turn causes termination of the calcium transient and repolarization of the VC neuron.

### VC inhibits HSN in the short term

**Figure 4:**
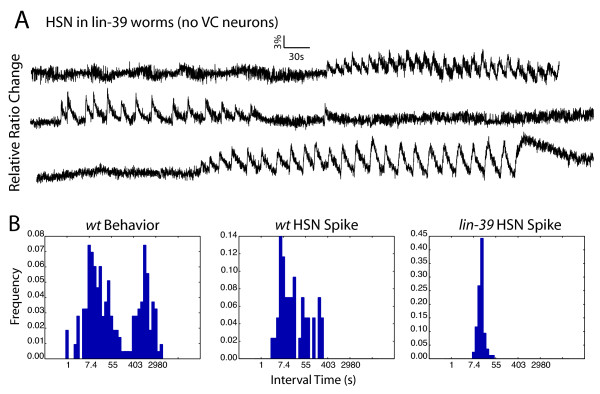
**VC inhibits HSN for short periods**. A) In *lin-39 *worms, where VC neurons are genetically ablated, the Ca^2+ ^level in HSN neurons alternates between oscillating and being silent, with all the short silent periods observed in wild-type HSN activity (less than several minutes in magnitude) having disappeared (three representative traces are shown). B) Histogram of wild-type egg-laying events, wild-type HSN spikes and *lin-39 *HSN spikes. It can be seen that without VC cells, HSN spike intervals longer than one minute are dramatically decreased.

Previous work indicated that the VC motorneurons inhibit the activity of the HSNs. To learn more about this interaction, we investigated the effect of eliminating the VCs on the dynamics of HSN activity in more detail. We observed that in worms which lack VC neurons because of a *lin-39(n709) *allele [4], single Ca^2+ ^spikes in HSN neurons are replaced by oscillatory trains, which are interspersed by very long silent periods. The distribution of short intervals appears sharper than in wild-type animals, suggesting a shift from the Poisson distribution (Figure 4B). Further statistical tests confirmed that the short inter-spike intervals in *lin-39 *animals follow a normal distribution comparable to the pattern observed when the HSN neuron is not receiving any inhibitory input. This implies that the VC neurons are mediating short-term inhibitory effects on HSN activity within the active state.

The VC neurons send arborized processes to the surface of the vulval epithelium in addition to their synapses on the vulval muscles. Based on the anatomical arrangement, it can be hypothesized that the function of the processes reaching the vulval epithelium is to sense stretch. Thus, VC neurons are suitably positioned to directly register a successful egg-laying event by sensing muscle or vulval epithelial stretch, and feed this information back to the HSN neurons by inhibiting their activity. Therefore, we hypothesize that individual egg-laying events might be detected by the VC neurons, which in turn provide short-term negative feed back to the HSNs to modulate their activity during the active state.

### Long-term feedback may be mediated by the uv1 cells

While the VC neurons appear to provide important inhibitory feedback to the HSN cells, additional cells are likely to provide further modulatory input to the egg-laying circuit. In *lin-39 *worms, in which the VC neurons are absent, very long silent periods (more than several minutes) are still observed between trains of HSN activity (Figure 4A). This observation suggests that another there may be additional, VC-independent inhibitory inputs to the HSNs that might act on longer time scales [5].

**Figure 5:**
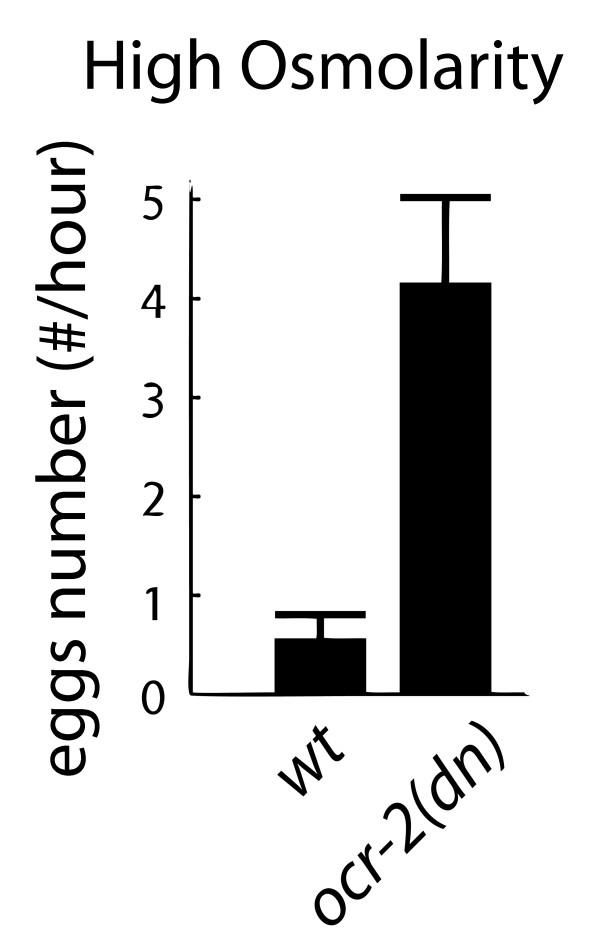
**uv1 cells might inhibit HSN for long periods when activated by a cluster of egg-laying events**. A) In *ocr-2(vs29) *worms, where uv1 cell activity is silenced due to an ion channel deficiency, the inhibitory effect of high osmolarity on egg-laying is lost.

Possible candidates for providing such inhibition are the uv1 neurosecretory cells [5]. These cells are located at the junction between the vulva and uterus, and have been shown to express neuropeptides as well as the catecholamine neurotransmitter tyramine [6,7]. They have been proposed to be potential touch sensors, based on their morphology and their expression of candidate sensory transduction molecules [5]. In wild-type worms, low external osmolarity causes constitutive egg-laying and continuous high-frequency calcium oscillations in HSN, while high osmolarity inhibits egg-laying and silences HSN calcium transients [3]. In *ocr-2(vs29) *mutant worms, where uv1 function is impaired due to a dominant negative missense mutation in a TRPV channel protein, egg-laying in high osmolarity conditions occurs at extremely high frequency [5], comparable to wild-type worms in low-osmolarity conditions (Figure 5). This suggests that the uv1 s may provide a long-term inhibitory input to the egg-laying circuit, and is consistent with a role for the uv1 s in mediating egg-laying inhibition by high osmolarity.

Since the uv1 s are candidate stretch receptor cells [5], we propose that they might be activated by movements of the vulva associated with egg-laying. In particular, they might facilitate the long quiescent periods that follow clusters of egg-laying events. Since the uv1 s release peptides and amines in a paracrine rather than a synaptic fashion, they may be well-suited to mediate long-lasting inhibition following multiple egg-laying events. Neurohormones have a much slower action and clearance kinetics compared to neurotransmitters released at synapses and this slow kinetics means that closely timed repeated neurotransmitter releasing events could give rise to an additive effect, building up a strong enough extracellular gradients to act at a longer distance. We therefore hypothesize that the uv1 s release an inhibitory neurohormone in response to a cluster of egg-laying events, inducing long term silencing of HSN activity and egg-laying behavior corresponding to the inactive phase of egg-laying.

### Circuit model of egg-laying pattern generator

**Figure 6:**
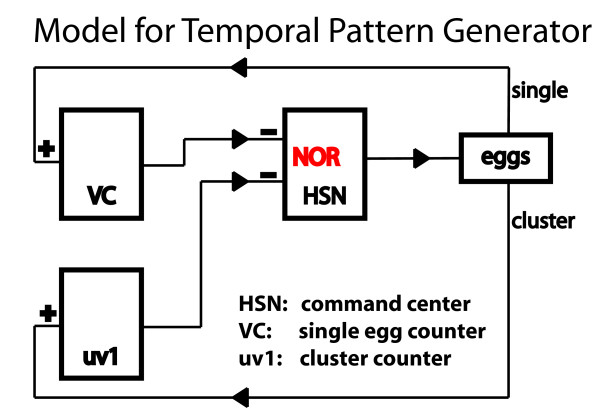
**A neuronal circuit model that produces a clustered temporal pattern in egg-laying behavior**. Details are discussed in the main text.

To summarize, our model of the clustered temporal pattern in egg-laying is as follows: (1) The HSN cells are the command neurons for egg-laying with individual Ca^2+ ^spikes corresponding to one egg-laying event; (2) the VC cells work as "single egg counters" that are briefly and transiently activated during individual egg-laying events and as a result inhibit HSN activity for short periods (about 20 seconds on average); and (3) the uv1 cells are the "cluster counters", activated by a cluster of egg-laying events and inhibiting HSN activity for longer periods (about 20 minutes on average). In our model, the VC and uv1 cells form two negative feedback loops sensing egg-laying behavior and feeding back onto HSN activity; however, they are different in their physiological activation mechanisms, and, when activated, differ in their kinetics of inhibiting HSN activity. Together, they generate a clustered temporal pattern as observed in natural egg-laying behavior. Figure 6 summarizes the resulting model. The corresponding mathematical equations are supplied in Additional Files 1 and 2. Please note that our model elucidates the cellular circuit connectivity, but does not propose a specific molecular mechanism, e.g., how HSN activity returns to the spiking state after being inhibited, or how VC quickly repolarizes HSN activity. This is due to the fact that our model is based on Ca^2+ ^dynamics, revealing only cell-level activities, not more detailed molecular mechanisms.

## Discussion

In our model, the HSN neurons can be seen as an information integration center, processing signals from uv1 and VC cells on top of their own autonomous activity. HSN functions as a digital 'NOR' gate, giving the output of "1" only if both VC and uv1 are inactive. The 'NOR' gate is the most universal logical gate in that it can be used to create all other 15 elementary logical gates (only the 'NAND' gate has the same universality). This means that even the most complex computers (and brains?) can be constructed based solely on a combination of 'NOR' gates [8]. The HSN egg-laying circuit is potentially an elegant example of a biological implementation of a 'NOR' calculation. It will therefore be a valuable system for more detailed studies of this mechanism, which may have intriguing implications in general biological computations.

This model immediately suggests some new characteristics of the temporal pattern that may be worthy of future investigation. According to our model, the number of eggs per cluster will be maintained at a relatively constant level, as it directly controls the switching off of the active state. Furthermore, if our model is correct, the distance between the HSN and uv1 cells should influence the number of eggs laid per cluster, because the longer the distance, the more neurohormone releasing events are required to build up a high enough concentration of hormone to terminate the active phase. Interestingly, HSN cells are first born at the tail and migrate to a location close to the vulva, traveling almost half of the body length (~50 micron). By evolution, the final position of HSN might be naturally selected to produce a certain number of eggs per cluster to achieve the best strategy for long-term species survival. It will be interesting to see if different natural variants of *C. elegans *have different HSN-vulva distances.

Previously, the clustered temporal pattern in egg-laying was described mathematically as two Poisson processes with different time constants (τ_1 _= 1/λ_1 _and τ_2 _= 1/(p*λ_2_)) and a switching probability *p *[2]. The model we developed from biological evidence fits that mathematical description surprisingly well and suggests possible specific biological pathways controlling each parameter. The Poisson-like nature of both long and short interval distributions can be explained by the HSN cells randomly switching back to the egg-laying state after being inhibited by either the VCs or the uv1s; the intra- and inter-cluster time constants correspond to the kinetics of these two switching events, respectively. The switching probability *p *in the mathematical description corresponds to how many consecutive egg-laying events in a cluster are required to activate the uv1 pathway. The value of *p *is determined by the distance between the HSN and uv1 cells together with the diffusion constant of uv1 neurohormones. This could potentially be validated in morphogenesis mutants, where the distance between neurons is altered.

Recently it has been found that reversal behavior of worms can also be described as a sum of two exponential processes (unpublished observations; Srivistava N, Clark D, Samuel AD). Therefore, controlled autonomous switching might be is regular feature of many behaviors (e.g., alternation between rest and arousal). For *C. elegans *egg-laying, two two-cell circuits, uv1-HSN and VC-HSN, control the state alternation. It will be interesting to see whether or not equivalently structured circuits are employed in other behavioral control situations.

## Conclusions

In systems biology, the usefulness of a computational model is mainly determined by its predictive power. The cellular and molecular model we present here is the first one for generating a clustered pattern and so far the simplest to explain all available data, including our own extensive genetic manipulation results and behavior recordings. At the same time it makes a number of very concrete, testable predictions that can now be explored by further experimentation, using additional advanced technologies. For instance, we suggest using vulva developmental mutants, which are incapable of accomplishing egg-laying, to confirm the existence of the postulated negative feedback loops. Likewise, the activity of the uv1 cells can be imaged using cameleon Ca^2+ ^indicators to see whether or not they are activated by egg-laying events. If the VCs directly sense egg-laying events, they are predicted to contain touch sensitive channels, which could be identified by electrophysiological studies. Finally, we predict that the egg-number per cluster is determined by the diffusion distance between HSN and the uv1 cells, which can again be tested in mutants or ablated animals. Testing concrete predictions like these will lead to a refined understanding of the molecular and cellular circuitry underlying the complex behavior of this important neurobiological model animal.

## Methods

### General methods

All nematodes were grown at 20.5+0.5°C on standard Nematode Growth Medium (NGM) seeded with *E. coli *strain OP50 as the food source. Nematodes were assayed 24 hours after late L4 stage at 20.5 ± 0.5°C.

### Egg-laying behavior assays

M9 egg-laying assays were performed in 96-well plates. 100 μl of melted 2% agarose in M9 were placed in each well and allowed to dry for 1-1.5 hours before starting the behavioral assay. Individual worms were placed on the solid agarose pad in each well and after about 1 min were covered with 50 μl of M9. The number of laid eggs was counted after 1.5 hours.

### Ca2+ imaging experiments

The protocol is the same as described in [3]. Combined fluorescent and visible microscopy was used to view both the Ca^2+ ^signal and egg-laying events simultaneously. The microscope equipment was as described earlier [9]; briefly, a Zeiss Axioskop 2 upright microscope equipped with a Hamamatsu Orca ER CCD camera, a Hamamatsu W-View emission image splitter and a Uniblitz Shutter (Vincent Associates) were used. A minimum of fluorescent light, 20-60% of the original light power with the neutral density filter (ND) of 2.0, was used. Weak visible light, barely revealing egg-laying events, was used to minimize interference with the cameleon fluorescent signal. Fluorescent images were acquired and saved using MetaVue 4.6 (Universal Imaging) at a frequency of either 33 Hz or 10 Hz for muscle and neuron imaging respectively, using a 63× Zeiss Achroplan water immersion objective.

Worms were immobilized with Nexaband S/C cyanoacrylate veterinary glue on a small agarose pad with the buffer of choice freshly made on a microscopy slide. The worms were then quickly covered with the buffer of choice and immediately moved under the microscope for recording. For HSN and vulval muscle imaging, the agarose pad was made in the same buffer as the bath. For VC imaging, a 2% agarose pad in M9 was used, regardless of the bath buffer, due to the fast adaptation of VCs to low osmolarity conditions. Agarose pads were made immediately before slide preparation in order to minimize the loss of focus in long recordings (10 mins), which usually is caused by swelling of the agarose pad. Worms were allowed to equilibrate for 2 to 4 mins before the start of recording.

## Authors' contributions

MZ designed and performed the experiments and drafted the manuscript. WRS initiated and supervised the study. RB contributed to the interpretation of data and helped to draft the manuscript. All authors read and approved the final manuscript.

## Supplementary Material

Additional File 1 **Details of the computational model**. PDF file listing the equations and parameter values used in the computational model described in Additional File 2. Click here for file

Additional File 2 **Computational model of the egg-laying circuit**. R file implementing the computational model of egg-laying described in the main text. Click here for file

## References

[B1] Norris BJ Weaver AL Morris LG Wenning A Garcia PA Calabrese RL A central pattern generator producing alternative outputs: temporal pattern of premotor activity J Neurophysiol 2006 96 1 309 326 10.1152/jn.00011.2006 16611849

[B2] Zhou GT Schafer WR Schafer RW A three-state biological point process model and its parameter estimation IEEE Trans on Signal Processing 1998 46 2698 2707 10.1109/78.720372

[B3] Zhang M Chung SH Fang-Yen C Craig C Kerr RA Suzuki H Samuel AD Mazur E Schafer WR A self-regulating feed-forward circuit controlling C. elegans egg-laying behavior Curr Biol 2008 18 19 1445 1455 10.1016/j.cub.2008.08.047 18818084 PMC2621019

[B4] Chalfie M Thomson JN Organization of neuronal microtubules in the nematode Caenorhabditis elegans J Cell Biol 1979 82 1 278 289 10.1083/jcb.82.1.278 479300 PMC2110421

[B5] Jose AM Bany IA Chase DL Koelle MR A specific subset of transient receptor potential vanilloid-type channel subunits in Caenorhabditis elegans endocrine cells function as mixed heteromers to promote neurotransmitter release Genetics 2007 175 1 93 105 10.1534/genetics.106.065516 17057248 PMC1774992

[B6] Alkema MJ Hunter-Ensor M Ringstad N Horvitz HR Tyramine Functions independently of octopamine in the Caenorhabditis elegans nervous system Neuron 2005 46 2 247 260 10.1016/j.neuron.2005.02.024 15848803

[B7] Kim K Li C Expression and regulation of an FMRFamide-related neuropeptide gene family in Caenorhabditis elegans J Comp Neurol 2004 475 4 540 550 10.1002/cne.20189 15236235

[B8] Kurzweil R The age of intelligent machines 1990 Cambridge, Mass; London: MIT Press

[B9] Suzuki H Kerr R Bianchi L Frokjaer-Jensen C Slone D Xue J Gerstbrein B Driscoll M Schafer WR In vivo imaging of C. elegans mechanosensory neurons demonstrates a specific role for the MEC-4 channel in the process of gentle touch sensation Neuron 2003 39 6 1005 1017 10.1016/j.neuron.2003.08.015 12971899

