# Supplementary material for: A circuit model of the temporal pattern generator of Caenorhabditis egg-laying behavior
Source: BMC Syst Biol. 2010 Jun 7;4:81. doi: 10.1186/1752-0509-4-81 (PMC2887794; doi:10.1186/1752-0509-4-81)
Supplement: Additional File 1 — Details of the computational model. PDF file listing the equations and parameter values used in the computational model described in Additional File 2. [file 1752-0509-4-81-S1.PDF]

A circuit model of the temporal pattern generator of *Caenorhabditis* egg-laying behavior Supplemental Material

Mi Zhang
William R. Schafer
Rainer Breitling

December 3, 2009

1 Equations

The following stochastic equations can be used to simulate the time course of egg-laying according to our circuit model:

$$p_0 = e^{-\tau/T_{1/2}} \tag{1}$$

$$p_1 = p(vc_{t+1} = 1|vc_t = 0) = \begin{cases} 1, & \text{if } egg_t = 1 \\ 0, & \text{otherwise} \end{cases} \tag{2}$$

$$p_2 = p(vc_{t+1} = 0|vc_t = 1) = \lambda_1 \tag{3}$$

$$p_3 = p(uv_{1,t+1} = 1|uv_{1,t} = 0) = \begin{cases} 1, & \text{if } count_t > 3 \\ 0, & \text{otherwise} \end{cases} \tag{4}$$

$$p_4 = p(uv_{1,t+1} = 0|uv_{1,t} = 1) = \lambda_2 \tag{5}$$

$$p_5 = p(hsn_{t+1} = 1) = \begin{cases} 1, & \text{if } uv_{1,t} + vc_t = 0 \\ 0, & \text{otherwise} \end{cases} \tag{6}$$

$$p_6 = p(egg_{t+1} = 1|egg_t = 0) = \begin{cases} 1, & \text{if } hsn_t = 1 \\ 0, & \text{otherwise} \end{cases} \tag{7}$$

$$p_7 = p(egg_{t+1} = 0|egg_t = 1) = 1 \tag{8}$$

$$count_{t+1} = p_0 * count_t + egg_t; \tag{9}$$

The variable $count_t$ describes the time-dependent concentration of neurotransmitter accumulating as a result of egg-laying events.

2 Parameters

The constant probability p_0 characterizes the clearance of neurotransmitter released from uv1 following an egg-laying event and therefore determining the average size of the clusters. It depends on the half-clearance time $T_{1/2}$, which is approximately 140 sec, and the time step size of the algorithm ($\tau = 0.5$ sec in the simulation shown below).

$$p_0 = e^{-\frac{ln(2)*\tau}{T_{1/2}}} = e^{-0.5*ln(2)/140} = 0.9975$$

The time constants λ_1 and λ_2 correspond to egg-laying intervals induced by vc and uv1 respectively. They can be estimated from the experimental data as described in Zhou et al. (1998). For the simulations of wild type egg laying we used:

$$\lambda_1 = \frac{1}{23} sec^{-1}; \lambda_2 = \frac{1}{1800} sec^{-1}$$

3 Simulations

The following figures illustrate real egg-laying data compared to a simulation of the model. For the simulation, the model was implemented in the statistical language R (R Development Core Team (2008). R: A language and environment for statistical computing. R Foundation for Statistical Computing, Vienna, Austria. ISBN 3-900051-07-0, URL http://www.R-project.org). The corresponding script is also included in the **Supplementary Material**. Experimental manipulations of the system, corresponding to the cell ablations and knock-outs used in our study, are easily introduced by manipulating the transition probabilities in the equations.

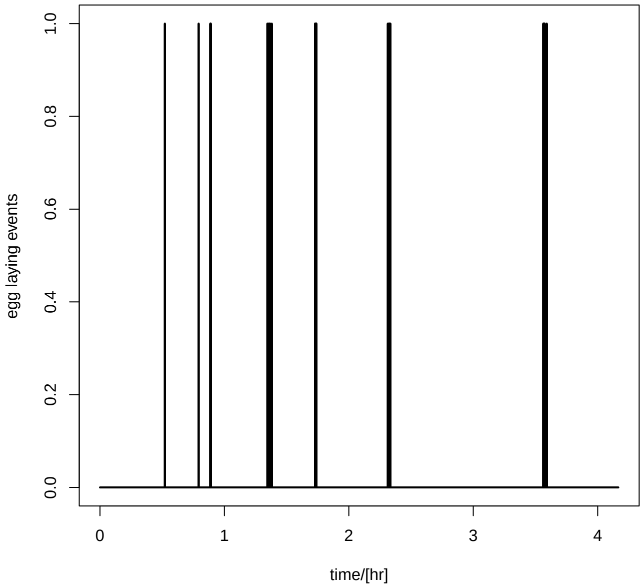

Figure 1: Experimentally observed egg-laying data.

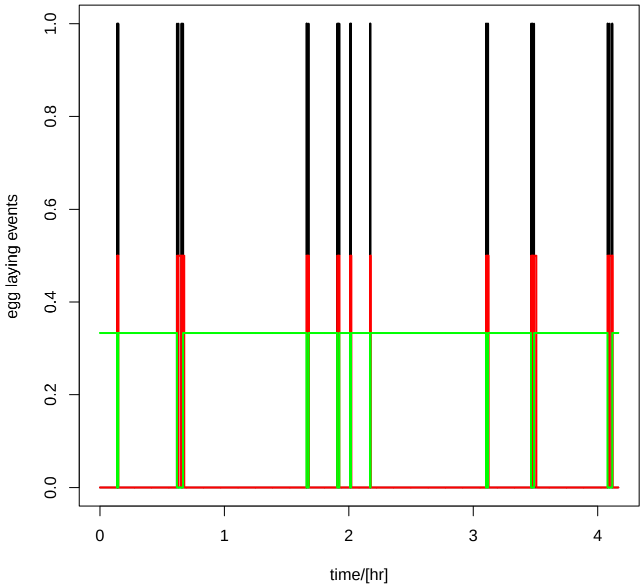

Figure 2: Simulated egg-laying data. The black lines here and in the previous figure correspond to individual egg-laying events, the red and green lines indicate the short and long periods of inhibition by vc and uv_1 neurons, respectively.
